# FRAX® Fracture Risks Are Associated with Coronary Artery Calcification Score

**DOI:** 10.1155/2017/1592598

**Published:** 2017-12-20

**Authors:** Tzyy-Ling Chuang, Yi-Da Li, Fu-Tsung Hsiao, Mei-Hua Chuang, Yuh-Feng Wang

**Affiliations:** ^1^Department of Nuclear Medicine, Dalin Tzu Chi Hospital, Buddhist Tzu Chi Medical Foundation, Chiayi, Taiwan; ^2^School of Medicine, Tzu Chi University, Hualien, Taiwan; ^3^Institute of Medical Sciences, College of Medicine, Tzu Chi University, Hualien, Taiwan; ^4^Department of Cardiology, Dalin Tzu Chi Hospital, Buddhist Tzu Chi Medical Foundation, Chiayi, Taiwan; ^5^Department of Medical Imaging, Dalin Tzu Chi Hospital, Buddhist Tzu Chi Medical Foundation, Chiayi, Taiwan; ^6^Department of Pharmacy, Dalin Tzu Chi Hospital, Buddhist Tzu Chi Medical Foundation, Chiayi, Taiwan

## Abstract

**Purpose:**

To examine the association between fracture risk assessment tool (FRAX) scores and coronary artery calcification (CAC) score in adults.

**Methods:**

The medical records of 81 adults who underwent both coronary computed tomography and bone mineral density (BMD) studies in a package during their health exams were reviewed at a regional hospital in Southern Taiwan. Data collected included health history, anthropomorphic characteristics, clinical laboratory results, and BMD. Fracture risk was determined using FRAX. Univariate and multivariate linear regression analysis were used to assess the association between CAC score and 10-year probability of hip fracture (HF) and major osteoporotic fracture (MOF) determined by FRAX.

**Results:**

The mean age of the patients was 55.8 years, and 63.0% were male. Univariate linear regression analysis showed that increases in MOF and HF risks, as measured by FRAX, were significantly and positively associated with CAC score. Multiple linear regression analysis adjusting for potential confounders showed that CAC score remained significantly associated with four FRAX indicators, including right MOF (*r* = 0.45, *P* < 0.001), left MOF (*r* = 0.31, *P* = 0.021), right HF (*r* = 0.38, *P* = 0.001), and left HF (*r* = 0.23, *P* = 0.049).

**Conclusions:**

Increased risks of MOF and HF as determined by FRAX were significantly and independently associated with CAC score.

## 1. Introduction

Osteoporosis and atherosclerosis frequently occur concomitantly, may share similar pathogenic mechanisms, and could be biologically linked [1, 2]. Fragility fractures are associated with a high risk of cardiovascular events, and patients with cardiovascular diseases have a higher fracture risk [3–5]. Severe abdominal aortic calcification is associated with higher cardiovascular morbidity and mortality, lower bone mineral density (BMD), and higher bone fragility and risk of fracture [6–9]. The link was significant for major fracture types, but data were less consistent for various fractures analyzed jointly [1, 10].

Coronary artery calcification (CAC) score is a surrogate marker for total calcified plaque burden and may predict future coronary events [11, 12]. An increased CAC score and subclinical atherosclerosis plaque burden as determined by multidetector row computed tomography (MDCT) is associated with a low BMD in all women, independent of cardiovascular risk factors and age [13].

The fracture risk assessment tool (FRAX), combining BMD and clinical risk factors to provide a comprehensive osteoporotic fracture risk assessment, may serve as a general guideline for the clinical management of osteoporosis [14]. Clinical risk factors used in FRAX include age, sex, weight, height, a prior history of fracture, use of oral glucocorticoids, current smoking, alcohol intake of three or more units daily, rheumatoid arthritis, a parental history of a hip fracture, and other secondary causes of osteoporosis. FRAX effectually estimates the 10-year likelihood of hip and major osteoporotic fractures.

The association of BMD or osteoporosis and other calcifications, such as coronary artery, carotid artery, and cardiac valve calcifications, has been reported, but with a paucity of clinical data. Further evaluation from the BMD value to the fracture risk is another issue. The purpose of this study was to explore the relationship of the 10-year probability of a hip fracture (HF) and a major osteoporotic fracture (MOF) as calculated with the FRAX and CAC scores.

## 2. Subjects and Methods

### 2.1. Subjects

We enrolled all clients who had received health examinations at the preventive medical center of a regional teaching hospital in Southern Taiwan between May 2014 and November 2015 to this study. A retrospective medical record review was performed. Those who were diagnosed with coronary arterial diseases; received coronary arterial catheter procedure, bypass surgery, and major heart operation; or followed with a history of bone fracture were all excluded. In addition, subjects need to receive both coronary CT scans and BMD tests during their health examination. This retrospective study was approved by the ethics committee of our institution, which approved waiver of informed consent from each patient. Medical records revealed personal information as follows: (1) comorbidities, such as hypertension, diabetes mellitus, and hyperlipidemia; (2) parent fractured hip history, current smoking, glucocorticoid usage, rheumatoid arthritis, secondary osteoporosis, and alcohol consumption; (3) anthropomorphic characteristics (age, sex, height, weight, and body mass index (BMI)); and (4) clinical laboratory findings including total cholesterol (TC), low-density lipoprotein cholesterol (LDL-C), high-density lipoprotein cholesterol (HDL-C), triglycerides (TG), glucose level, systolic blood pressure (SBP), and diastolic blood pressure (DBP).

### 2.2. Coronary Artery Calcification Score

CAC score was obtained from unenhanced axial images scanned prior to coronary CT angiography. The scans were performed using a multidetector CT system (LightSpeed VCT, GE Medical Systems). CAC was quantified with the Agatston scoring method, as previously described [15]. Total calcium score was determined using the sum of individual scores for the four major coronary arteries (left main, left anterior descending, circumflex, and right coronary arteries).

### 2.3. Bone Mineral Density

BMD was assessed by dual-energy X-ray absorptiometry (DXA) using a Discovery Wi DXA system (Hologic Inc.). Absolute BMD values and T-scores (number of standard deviations below the BMD of a young normal reference group, Asia database) were calculated for all patients. The measured areas included the lumbar spine and bilateral hips (total and (femoral) neck regions). The same densitometer was used for all patients in order to ensure accurate comparisons.

### 2.4. FRAX Calculations

The 10-year probability (expressed as a percentage) of HF and MOF were calculated for all subjects. All fracture risk factors included in the FRAX (age, sex, weight, height, previous fracture history, parent fractured hip history, current smoking, glucocorticoid usage, rheumatoid arthritis, secondary causes of osteoporosis, and alcohol intake of 3 or more units/day) were assessed, as well as right and left hip femoral neck BMD data. The FRAX score was calculated on country-specific (Taiwan) data on the website (https://www.shef.ac.uk/FRAX/tool.aspx?lang=en).

### 2.5. Statistical Analysis

Results were expressed as mean ± standard deviation, or number (percentage), as appropriate. Differences in means or frequencies were tested by chi-squared test or *t*-test, as appropriate. Simple linear regression analysis was performed with CAC values as the dependent variable. Clinical characteristics, laboratory data, BMD, and FRAX were the independent variables. Multiple linear regression models for FRAX were performed and adjusted for age, sex, TC, LDL, HDL, TG, SBP, and DBP. All statistical analyses were performed using the PASW Statistics 18 suite (SPSS Inc., Chicago, IL).

## 3. Results

### 3.1. Subject Characteristics

Demographic and clinical characteristics of subjects are presented in Table 1. The study population was predominantly male (63%), with a mean age of 55.8 ± 9.9 years. Analysis of clinical characteristics indicated that 23.5% of patients had hypertension, 11.1% were diabetic, 6.2% had hyperlipidemia, 7.4% were smokers, 17.3% had risk factors of secondary osteoporosis, and 4.9% drank alcohol. There were significant differences between male and female subjects as to height, weight, BMI, lipid profile, and DBP.

### 3.2. Bone Mineral Density

Mean lumbar spine T-scores on DXA were −0.33 ± 1.16 for men, −1.12 ± 1.14 for women, and −0.63 ± 1.21 for all patients. There were significant differences between male and female in the BMD values of the lumbar spine, femoral neck, and total regions of the bilateral hips. Spine and hip T-scores were significantly different between male and female, except the T-score of the total region of the bilateral hips (Table 1).

### 3.3. CAC Score Risk Factors

The mean CAC score of all subjects was 123.8 ± 294.2 (range, 0–1771). Simple linear regression analysis showed that CAC score correlated positively with hypertension (*r* = 0.58, *P* < 0.001), hyperlipidemia (*r* = 0.42, *P* < 0.001), TG (*r* = 0.37, *P* = 0.001), and SBP (*r* = 0.22, *P* = 0.048) (Table 2). Right hip femoral neck T-score and left hip total area T-score showed significant inverse correlation with CAC score (*r* = −0.25, *P* = 0.027; *r* = −0.30, *P* = 0.007) (Table 2).

### 3.4. FRAX and CAC Score

The FRAX scores for MOF risks of the bilateral hips showed a significant difference between male and female (Table 1). Univariate linear regression analysis showed that CAC score correlated positively with MOF and HF risks of the right hip (*r* = 0.32, *P* = 0.003; *r* = 0.31, *P* = 0.005) and MOF and HF risks of the left hip (*r* = 0.26, *P* = 0.021; *r* = 0.23, *P* = 0.036) as calculated by FRAX (Table 2). The positive relationships for all MOF and HF risks determined by FRAX remained in the multiple linear regression models after being adjusted for age, sex, TC, LDL-C, HDL-C, TG, SBP, and DBP (Table 3).

## 4. Discussion

Hip fractures are associated with higher risk of myocardial infarction [16]. Patients with cardiovascular diseases have a higher risk of major osteoporotic fractures [5]. Patients with recent coronary events have a higher prevalence of vertebral fractures, which is independent of BMD [17]. CAC scores measured by MDCT can be used as a marker of subclinical and overt atherosclerosis and predicts future cardiovascular events independently of other risk factors [11, 12]. In our analysis using simple linear regression, we found that similar to prior studies, hypertension, hyperlipidemia, BP, and TG were positively correlated with CAC score and with risk for coronary as well as other cardiovascular events [18, 19].

A 2009 study which focused on the coronary artery found that the BMD of the femur and lumbar spine were negatively associated with the CAC score after adjusting for age and metabolic parameters in women, but not in men [13]. In their study, the correlation between BMD and CAC score was more significant in the femur area than in the lumbar spine. In our study, only the right femoral neck T-score and left total hip T-score showed a significantly inverse relationship with the CAC score. We also found that fracture risks, rather than merely BMD, were significantly positively correlated with CAC scores. This is probably because, in addition to the BMD value, the calculation of MOH and HF risks takes into account other fracture risk factors. Recently, vascular calcification promoters and inhibitors have been identified [2]. Cell-culture study has demonstrated that oxidized low-density lipoprotein-cholesterol (LDL-C) can inhibit the differentiation of osteoblasts in bone, as in osteoporosis, and promote calcification of smooth muscle vascular cells, as in atherosclerosis [20]. Calcification of arteries involves genetic factors, hormones, cytokines, abnormal mineral metabolism, transport of calcium and phosphate, transdifferentiation of vascular smooth muscle cells towards an “osteoblast-like” phenotype, and other factors [21]. Bone loss, and subsequently increased fracture risk and then fractures, and atherosclerosis are linked processes. If fragility fractures of bone and vascular calcification have reciprocal causation and are mutually related, their involved, dynamic, highly regulated process in the physiologic microenvironment of bone and vessels may provide a more reasonable explanation for this linkage.

Our results indicated that the FRAX can help identify individuals who are at high risk of osteoporotic fractures and at higher risk of an increased CAC score, as well as cardiovascular events. In this study, adjusting for age, sex, TC, LDL-C, HDL-C, TG, SBP, and DBP did not attenuate the associations between MOF or HF risks and CAC severity. In our study, with consideration of a possible altered disease status and physiological condition after treatment, one model was adjusted with the lipid profile and BP, but not with the comorbidities. Thus, FRAX can be used to predict CAC severity and identify individuals in whom early treatment may prevent future fractures and cardiovascular events.

There were several limitations to this study. First, this was a retrospective and observational study with a relatively small sample size. We did not measure serum markers which may be possible mechanisms for the link between coronary CAC, osteoporosis, and bone fragility, such as bone resorption, estrogen, vitamin D, parathyroid hormone, calcium intake, thyroid-stimulating hormone, T3, free T4, or osteoprotegerin levels. We did not examine the results as compared to other fracture risk assessment tools, such as the osteoporosis self-assessment tool for Asians (OSTA), Garvan fracture risk calculator (GARVAN), osteoporosis risk assessment instrument (ORAI), osteoporosis index of risk (OSIRIS), and simple calculated osteoporosis risk estimation (SCORE).

## 5. Conclusions

Increased risks of MOF and HF as estimated by FRAX are significantly and independently associated with more severe CAC scores. DXA and FRAX can be used to predict fracture risk and CAC scores and identify patients who may benefit from early intervention.

## Figures and Tables

**Table 1:** Demographic and clinical characteristics of study participants.

	All	Male	Female	*P* value
Number	81	51	30	
Age (range) (years)	55.8 ± 9.9 (27–81)	55.1 ± 10.6 (27–81)	57.1 ± 8.6 (34–75)	0.379
Height (cm)	164.0 ± 8.2	168.3 ± 6.8	156.6 ± 4.2	<0.001
Weight (kg)	67.9 ± 12.3	73.1 ± 11.2	59.0 ± 8.5	<0.001
BMI (kg/m^2^)	25.1 ± 3.2	25.7 ± 3.0	24.1 ± 3.5	0.029
Smoking (%)	6 (7.4)	6 (11.8)	0 (0.0)	0.080
Drinking (%)	4 (4.9)	4 (7.8)	0 (0.0)	0.291
Hypertension (%)	19 (23.5)	10 (19.6)	9 (30.0)	0.286
Diabetes mellitus (%)	9 (11.1)	7 (13.7)	2 (6.7)	0.473
Hyperlipidemia (%)	5 (6.2)	3 (5.9)	2 (6.7)	>0.999
Secondary osteoporosis (%)	14 (17.3)	8 (15.7)	6 (20.0)	0.620
TC (mg/dL)	189.1 ± 32.7	183.1 ± 32.3	199.3 ± 31.3	0.031
LDL-C (mg/dL)	121.6 ± 27.5	116.8 ± 30.1	129.7 ± 20.4	0.025
HDL-C (mg/dL)	48.6 ± 15.0	43.7 ± 10.7	57.0 ± 17.5	<0.001
Triglycerides (mg/dL)	145.2 ± 95.5	166.0 ± 109.0	109.8 ± 51.1	0.002
Glucose (mg/dL)	107.4 ± 20.8	110.1 ± 24.5	102.8 ± 11.0	0.073
SBP (mmHg)	126.0 ± 22.9	127.2 ± 18.3	124.1 ± 29.4	0.606
DBP (mmHg)	78.7 ± 15.4	81.7 ± 15.4	73.7 ± 14.1	0.022
Lumbar spine BMD (g/cm^2^)	0.969 ± 0.139	1.011 ± 0.129	0.898 ± 0.129	<0.001
Lumbar spine T-score	−0.63 ± 1.21	−0.33 ± 1.16	−1.12 ± 1.14	0.004
Right neck BMD (g/cm^2^)	0.732 ± 0.118	0.769 ± 0.123	0.669 ± 0.075	<0.001
Right neck T-score	−1.04 ± 0.95	−0.81 ± 1.00	−1.41 ± 0.74	0.006
Right total BMD (g/cm^2^)	0.838 ± 0.141	0.874 ± 0.128	0.778 ± 0.144	0.003
Right total T-score	−0.42 ± 0.93	−0.37 ± 0.84	−0.51 ± 1.08	0.492
Left neck BMD (g/cm^2^)	0.738 ± 0.122	0.772 ± 0.121	0.682 ± 0.105	0.001
Left neck T-score	−1.01 ± 0.99	−0.78 ± 1.00	−1.39 ± 0.87	0.007
Left total BMD (g/cm^2^)	0.840 ± 0.139	0.882 ± 0.118	0.768 ± 0.143	<0.001
Left total T-score	−0.41 ± 0.91	−0.30 ± 0.83	−0.59 ± 1.01	0.166
Right MOF (FRAX) (%)	4.84 ± 2.87	4.03 ± 2.18	6.21 ± 3.37	0.003
Right HF (FRAX) (%)	1.39 ± 1.62	1.27 ± 1.59	1.60 ± 1.68	0.379
Left MOF (FRAX) (%)	4.88 ± 3.11	3.99 ± 2.35	6.39 ± 3.66	0.002
Left HF (FRAX) (%)	1.41 ± 1.74	1.22 ± 1.69	1.73 ± 1.80	0.207
CAC score	123.8 ± 294.2	137.1 ± 315.0	101.1 ± 258.7	0.598

BMI: body mass index; TC: total cholesterol; LDL-C: low-density lipoprotein cholesterol; HDL-C: high-density lipoprotein cholesterol; SBP: systolic blood pressure; DBP: diastolic blood pressure; BMD: bone mineral density; MOF: 10-year probability of a major osteoporotic fracture; HF: 10-year probability of a hip fracture; FRAX: fracture risk assessment tool; TBS: trabecular bone score; CAC score: coronary artery calcification score. Data are expressed as mean ± standard deviation, unless otherwise specified.

**Table 2:** Simple linear regression analysis of coronary artery calcification.

	*R* ^2^	Coefficient	95% CI	*P* value	Std *β* (*r*)
Age (years)	0.045	6.27	−0.23–12.76	0.058	0.21
Sex (*M* = 1, *F* = 0)	0.004	35.97	−99.38–171.33	0.598	0.06
Height (cm)	0.005	−2.52	−10.52–5.48	0.532	−0.07
Weight (kg)	0.001	0.64	−4.71–6.00	0.812	−0.03
BMI (kg/m^2^)	0.008	8.35	−11.87–28.56	0.414	0.09
Smoking	0.002	−45.09	−294.91–204.73	0.720	−0.04
Drinking	0.007	−110.98	−412.17–190.21	0.465	−0.08
Hypertension	0.340	402.36	276.82–527.90	<0.001	0.58
Diabetes mellitus	0.011	99.57	−107.59–306.73	0.342	0.11
Hyperlipidemia	0.175	508.01	260.85–755.17	<0.001	0.42
Secondary osteoporosis	0.018	104.56	−67.03–276.15	0.229	0.14
TCH (mg/dL)	0.019	1.24	−0.75–3.24	0.219	0.14
LDL (mg/dL)	0.013	−1.20	−3.58–1.18	0.320	−0.11
HDL (mg/dL)	<0.001	0.01	−4.40–4.41	0.998	<0.001
Triglycerides (mg/dL)	0.136	1.14	0.50–1.78	0.001	0.37
Glucose (mg/dL)	<0.001	0.16	−3.01–3.33	0.921	0.01
SBP (mmHg)	0.049	2.83	0.02–5.63	0.048	0.22
DBP (mmHg)	0.023	2.92	−1.32–7.16	0.175	0.15
Lumbar spine BMD (g/cm^2^)	<0.001	2.21	−470.38–474.80	0.993	0.001
Lumbar spine T-score	0.002	−10.21	−64.60–44.18	0.710	−0.04
Right neck BMD (g/cm^2^)	0.044	−524.47	−1072.08–23.13	0.060	−0.21
Right neck T-score	0.060	−75.63	−142.53–8.74	0.027	−0.25
Right total BMD (g/cm^2^)	0.002	−102.99	−569.25–363.27	0.661	−0.05
Right total T-score	0.032	−56.90	−126.44–12.64	0.107	−0.18
Left neck BMD (g/cm^2^)	0.035	−451.74	−980.29–76.81	0.093	−0.19
Left neck T-score	0.046	−63.47	−128.25–1.31	0.055	−0.21
Left total BMD (g/cm^2^)	0.020	−303.22	−774.01–167.57	0.204	−0.14
Left total T-score	0.088	−96.33	−165.84–26.81	0.007	−0.30
Right MOF (FRAX) (%)	0.103	32.96	11.20–54.71	0.003	0.32
Right HF (FRAX) (%)	0.096	56.27	17.56–94.97	0.005	0.31
Left MOF (FRAX) (%)	0.066	24.34	3.85–44.84	0.021	0.26
Left HF (FRAX) (%)	0.055	39.59	2.74–76.44	0.036	0.23

M: male; F: female; BMI: body mass index; TC: total cholesterol; LDL-C: low-density lipoprotein cholesterol; HDL-C: high-density lipoprotein cholesterol; SBP: systolic blood pressure; DBP: diastolic blood pressure; BMD: bone mineral density; MOF: 10-year probability of a major osteoporotic fracture; HF: 10-year probability of a hip fracture; FRAX: fracture risk assessment tool; TBS: trabecular bone score; CAC score: coronary artery calcification score.

**Table 3:** Multiple linear regression analysis of coronary artery calcification for FRAX.

	*R* ^2^	Coefficient	95% CI	*P* value	Std *β* (*r*)
Right MOF (FRAX) (%)	0.381	46.55	21.45–71.65	<0.001	0.45
Right HF (FRAX) (%)	0.369	68.31	28.91–107.70	0.001	0.38
Left MOF (FRAX) (%)	0.316	29.22	4.44–53.99	0.021	0.31
Left HF (FRAX) (%)	0.302	39.10	0.15–78.04	0.049	0.23

Each of the nine separate multiple linear regression models was adjusted for age, sex, TC, LDL-C, HDL-C, TG, SBP, and DBP.
